# Morgan's Plastic Gold

**Published:** 1870-04

**Authors:** 


### ARTICLE X.

### *Morgan's Plastic Gold.*

At a late meeting of the Buffalo City Dental Association, the subject of "Filling approximal cavities in bicuspids" came up for discussion. The debate was one of considerable interest to those present, but did not present originality enough to be worthy of a full report. One point, perhaps, however, may be of interest to some of our readers. We refer to the practicability of anchoring the gold in the cavity without using retaining points. This can be done by the use of Morgan's Plastic Gold. Other Sponge Golds may answer the same purpose, but in the hands of the writer, the Plastic Gold has been more manageable than other kinds. If the floor of the cavity is covered with this gold, freshly annealed, and it is then thoroughly condensed, making the pressure directly perpendicular to the floor of the cavity, it can be carried down and anchored, so that further additions of gold can be made without danger of causing any "rocking" or dislodgment. In condensation, the plugger will appear to carry down just what gold is before it, without dragging or disturbing the surrounding mass, and the gold will cohere to the slightly roughened dentine sufficiently to retain its place. The writer uses the Automatic Plugger thoroughly upon the first layer, and then proceeds to build up the remainder of the filling with foil. One point further requires attention. In approximal cavities, the Plastic Gold should not be permitted to approach the edge of the cavity at any point, especially not at the cervical wall. Many fillings excellent in all other respects, have proved defective from this cause, as experience has shown that the entire part of the filling which it is essential should form a water-tight joint, should be made of foil. The *Dental Times* for January, 1868, contains a report on Plastic Gold, read before the Pennsylvania Association of Dental Surgeons, which explains the reason of this precaution. The whole report is well worthy of study, but from our limited space, we can only append a few extracts. Fillings of both Plastic Gold and Foil were made in a block of steel, so arranged as to allow of the easy removal of the plug, and were examined under the microscope.

"A filling of Lamm's Gold was carefully packed by a member of the committee, who spent much time in endeavoring to secure a perfect filling. The microscopical examination of this filling exhibited an an external surface equally as good as foil. The periphery of the filling, at the edge of the cavity, was equally perfect with the center, the crystalline structure being entirely removed. This, how ever, the operator reports was not attained without a large expenditure of time. The gold at the bottom of the cavity did not present the compact structure that the foil filling exhibited, made under similar circumstances and in the same cavity, the crystals being almost as prominent as in unworked gold."

Among the conclusions arrived at were:

"That if sufficient labor be bestowed upon Plastic Gold, a surface equal to the gold foil can be made. But this can only be accomplished at the periphery of the filling by an amount of labor largely in excess of that required foil in similar positions."

These experiments were conducted by as good operators as there are in the country, and the results are worthy of the attention and acceptance of the dentist. A few dentists of superior ability, have used sponge gold exclusively, and have made for themselves a well deserved reputation for the excellence of their operations. Indeed, attention was first generally called to the building up and restoration of the shape of the teeth, by the use of sponge gold; but while a few have achieved success, the greater number of those who have attempted its use have abandoned it, and again taken up the use of foil. But while Plastic Gold may be regarded as inferior to foil as a material for fillng teeth, it will be found invaluable for overlaying the floor of the cavity, if dentists will become habituated to its use for that purpose. To obtain entirely satisfactory results, enough of it must be used to make its properties fully understood, but when this point is reached, the dentist will find one of his most tedious and vexatious obstacles removed.

It should first be used in crown cavities, until some degree of dexterity is acquired in the manipulation of the gold, and afterwards in places more difficult. The proper precautions should be taken to ensure dryness of the cavity. *Good* fillings cannot be made unless this point is fully appreciated and attended to.—*Dental Advertiser.*

---

